# Draft Genome Sequence of *Nocardioides luteus* Strain BAFB, an Alkane-Degrading Bacterium Isolated from JP-7-Polluted Soil

**DOI:** 10.1128/genomeA.01529-16

**Published:** 2017-01-26

**Authors:** Lisa M. Brown, Thusitha S. Gunasekera, Oscar N. Ruiz

**Affiliations:** a University of Dayton Research Institute, Dayton, Ohio, USA; b Fuels and Energy Branch, Aerospace Systems Directorate, Air Force Research Laboratory, Wright-Patterson AFB, Ohio, USA

## Abstract

*Nocardioides luteus* strain BAFB is a Gram-positive bacterium that efficiently degrades C_8_ to C_11_ alkanes aerobically. The draft genome of *N. luteus* BAFB is 5.76 Mb in size, with 5,358 coding sequences and 69.9% G+C content. The genes responsible for alkane degradation are present in this strain.

## GENOME ANNOUNCEMENT

*Nocardioides luteus* strain BAFB is a Gram-positive, high G+C, aerobic actinomycete bacterium isolated from soil contaminated with JP-7, a light petroleum hydrocarbon distillate fuel, from Beale Air Force Base in Northern California. This strain was shown to degrade alkanes in the range of C_8_ to C_11_ efficiently (1). Our results confirmed that *N. luteus* BAFB exhibits efficient degradation of C_8_ to C_11_ normal and mono-branched alkane compounds. Several *Nocardioides* spp. have been isolated from environments contaminated with hydrocarbons and shown to degrade aromatics and alkanes (2 – 4). Based on BLAST analysis (http://blast.ncbi.nlm.nih.gov/Blast.cgi) of the 16S rRNA gene sequence, *N. luteus* BAFB is 100% similar in 98% of the sequence to *N. albus* KAUST1; 96% similar to *Nocardioides* sp. CF8 (5) and *N. nitrophenolicus* JCM 10703; and 94% similar to *N. aromaticivorans* JCM 11674. We have sequenced the genome of *N. luteus* BAFB to understand the mechanisms controlling its hydrocarbon-adaptation and alkane-degradation activities.

*N. luteus* BAFB was sequenced on an Illumina HiSeq platform using a whole-genome shotgun approach, producing 8,457,792 reads. Roche *de novo* assembly software (Newbler version 2.9) aligned the reads into 86 large (>500 bp) contigs with an average coverage of 111× and an *N*_50_ of 121,485 bp. The largest contig extended 261,750 bp. The draft genome sequence was 5,761,811 bp in length with a G+C content of 69.9%. The NCBI Prokaryotic Genome Annotation Pipeline (PGAP; https://www.ncbi.nlm.nih.gov/genome/annotation_prok) predicted 5,476 genes, including 5,358 coding sequences (CDSs), two rRNAs, 48 tRNAs, and 118 pseudogenes. Rapid genome annotations using the RAST server (6) assigned the coding sequences to 442 subsystems, of which amino acids and derivatives (*n* = 561 CDSs), carbohydrates (*n* = 492), cofactors, vitamins, prosthetic groups, and pigments (*n* = 328), protein metabolism (*n* = 286), fatty acids, lipids, and isoprenoids (*n* = 255), stress response (*n* = 138), metabolism of aromatic compounds (*n* = 119), respiration (*n* = 117), RNA metabolism (*n* = 111), DNA metabolism (*n* = 110), nucleosides and nucleotides (*n* = 127), and virulence, disease, and defense (*n* = 95) were most abundant.

The NCBI PGAP predicted a large number of oxygenases and hydrolases, including three predicted alkane monooxygenase genes (*alkB*), two with at least 76% homology to *Nocardioides* sp. CF8 and one with 78% similarity to *Streptomyces* sp. CdTB01. Also, genes for two methane monooxygenase subunits were observed. Twelve P450 genes, with one presenting 79% homology to the *Gordonia* sp. TF6 cytochrome P450 alkane hydroxylase, were predicted. P450 cytochromes were shown to be involved in the degradation of branched alkanes (7). Other important genes involved in the degradation of hydrocarbons and aromatic compounds include phenol monooxygenase, catechol 1,2-dioxygenase, homogentisate 1,2-dioxygenase, 2-nitropropane dioxygenase, benzoate 1,2-dioxygenase, alkanesulfonate monooxygenase, and dimethyl sulfone monooxygenase. The genes of the central protocatechuate catabolic pathway for aromatic degradation, including protocatechuate 3,4-dioxygenase, 3-carboxymuconate cycloisomerase, and 4-carboxymuconolactone decarboxylase, were present. However, this bacterium lacked key aromatic hydroxylating enzymes such as benzene 1,2-dioxygenase and naphthalene 1,2-dioxygenase, which are required for the degradation of benzene and naphthalene hydrocarbons, respectively. The *N. luteus* BAFB genome will facilitate the study of pathways and adaptive mechanisms leading to the degradation and bioremediation of recalcitrant hydrocarbon compounds.

### Accession number(s).

This project has been deposited at DDBJ/EMBL/GenBank under the accession number JZDQ00000000.
